# Magnetic Molecularly Imprinted Polymers: Synthesis and Applications in the Selective Extraction of Antibiotics

**DOI:** 10.3389/fchem.2021.706311

**Published:** 2021-08-04

**Authors:** Junyu Li, Yiran Wang, Xiuxia Yu

**Affiliations:** Department of Chemistry, Shandong University, Weihai, China

**Keywords:** antibiotics, magnetic molecularly imprinted polymers, detection, synthesis, polymerization

## Abstract

Recently, magnetic molecularly imprinted polymers (MMIPs) have integrated molecular imprinting technology (MIT) and magnetic separation technology and become a novel material with specific recognition and effective separation of target molecules. Based on their special function, they can be widely used to detect contaminants such as antibiotics. The antibiotic residues in the environment not only cause harm to the balance of the ecosystem but also induce bacterial resistance to specific antibiotics. Given the above consideration, it is especially important to develop sensitive and selective methods for measuring antibiotics in the complex matrix. The combination of MMIPs and conventional analytical methods provides a rapid approach to separate and determine antibiotics residues. This article gives a systematic overview of synthetic approaches of the novel MMIPs materials, briefly introduces their use in sample pretreatment prior to antibiotic detection, and provides a perspective for future research.

## Introduction

Antibiotics are widely available drugs that are employed to tackle bacterial infections in humans, animals, and plants (Thiele-Bruhn, 2003). The world's annual usage of antibiotics exceeds 100,000 tons (Danner et al., 2019). Due to the large-scale use of antibiotics, they are inevitably introduced into the environment, such as sewage effluents, surface waters, drinking water, and soil (Sarmah et al., 2006). The prevalence of antibiotics residues may induce the occurrence of antibiotic-resistant bacteria and antibiotic-resistant genes, increasingly becoming one of the most critical challenges facing all human societies worldwide (Qiao et al., 2018). In the United States, there are more than 2.8 million antibiotic-resistant infections each year, resulting in more than 35,000 deaths. In addition, according to conservative estimates, at least 12,800 people died from antibiotic-resistant bacteria (*Clostridioides difficile*) in 2017 (Centers for Disease Control and Prevention, 2019). Indeed, the first antibiotic resistance threat report published by the Centers for Disease Control and Prevention in 2013 served as a wake-up call for the danger of antibiotic resistance. Furthermore, the World Health Organization has also recognized antibiotic resistance as a serious threat to public health (World Health Organization, 2015). With the increasing problem of antibiotic resistance, there is an urgent need to look for efficient and accurate antibiotic determination methods to provide a scientific basis for the management and supervision of antibiotics.

Currently, various methods for antibiotic detection in complex environmental samples have emerged. They mainly include high-performance liquid chromatography, high-performance liquid chromatography-mass spectrometry, solid-phase liquid mass spectrometry, high-performance liquid chromatography fluorescence detection, microchip electrophoresis, gas chromatography, and liquid chromatography-mass spectrometry (Golet et al., 2001; Gantverg et al., 2003; Lindberg et al., 2004; Renew and Huang, 2004; Wang et al., 2006; Vass et al., 2008; Gros et al., 2013; Bosma et al., 2020; Wang et al., 2021). Nevertheless, these approaches associated with antibiotic detection have the following problems: tedious separation/clean-up procedures, longer analysis time, and severe matrix interference in complex samples (Schenck and Callery, 1998; Samanidou and Nisyriou, 2008). Besides, the performance of analytical instruments is closely related to the separation of samples (Ashley et al., 2017). To overcome these limitations, researchers are crying out for innovative sample pretreatment techniques to eliminate the matrix effect and improve detection selectivity. Molecularly imprinted polymers (MIPs) have been drawn scholars' immense attention.

MIPs are a class of specific affinity materials that possess identifying cavities corresponding to the shape, size, and functional group of template molecules (Chen et al., 2016; Zhou et al., 2019). MIPs can be obtained through conventional methods such as bulk polymerization, precipitation polymerization, and emulsion polymerization (Ding and Heiden, 2014). The synthesis of MIPs was based on a copolymerization reaction between template molecules and functional monomers via either non-covalent or covalent interactions. Subsequently, the monomers and crosslinking agents are polymerized together around the template to create a hyper-crosslinked polymer network. Selective binding sites are constructed by removing the template (Aeinehvand et al., 2017; Zhou et al., 2019). MIPs could be selected as a suitable candidate for the separation and preconcentration of complex matrices owing to their promising properties, such as low cost, ease of synthesis, excellent selectivity and sensitivity, exceptional reusability, and high tolerance to the experimental conditions (Chen et al., 2011; Lian et al., 2012; Ahmad et al., 2015; Luo et al., 2019).

MIPs have made significant progress recently; however, obvious drawbacks still exist during their practical use. In highly crosslinked polymers, the target molecules encapsulated in the polymers are difficult to elute due to higher viscosity, bringing about an apparent reduction in the selectivity and specificity of MIPs, subsequently influencing the performance of separation and purification (Zhang et al., 2019). MMIPs have greatly attracted the interest of researchers, as these materials can be considered a potential solution to aforementioned problems. MMIPs not only offer advantages of MIPs but also hold the characteristics of high dispersion stability, magnetic separation, and modifiable chemical surface (Chen et al., 2018). MMIPs can achieve rapid separation by an external magnetic field (Chen et al., 2013).

Currently, several approaches for the synthesis of MMIPs have been reported. The synthetic methods of MMIPs will be summarized in this article. Furthermore, MMIPs can provide a new solution for isolating and detecting antibiotic residues in complex matrix, so we set our sights on recent progress in the application of common antibiotics detection by MMIPs. Finally, we propose future perspectives of research direction.

## Synthesis of Magnetic Molecularly Imprinted Polymers

MMIPs can be obtained by various strategies; the common synthesis process generally consists of four steps. The first step is to synthesize magnetic nanoparticles (MNPs), the second step is the surface modification of magnetic components, the third step is polymerization with functional nanoparticle as magnetic core when template molecules, functional monomers, and crosslinker exist, and the fourth step is the removal of template molecules from the polymer (Chen and Li, 2012).

### Preparation of MNPs

A multitude of synthetic routes have been established for preparing MNPs, such as co-precipitation, thermal decomposition, microemulsion synthesis, solvothermal/hydrothermal synthesis, sol-gel synthesis, flow injection syntheses, and microwave-assisted synthesis (Salazar-Alvarez et al., 2006; Xia et al., 2011; Mahdavi et al., 2013; Tong et al., 2015; Dinc et al., 2019; Nguyen et al., 2020; Cervera et al., 2021; Mohammadi Badizi and Maleki, 2021; Saladino et al., 2021; Salvador et al., 2021). Co-precipitation and solvothermal methods are the most popular approaches to acquire high-quality MNPs.

### Co-Precipitation Synthesis

Co-precipitation mainly adds an alkaline substance to an aqueous salt solution containing Fe^2+^/Fe^3+^ under an inert atmosphere, generating iron oxide nanoparticles (IONPs) through a precipitation reaction. The method is an easily controlled preparation process, with low production costs, a short production cycle, and little environmental pollution.

The regulation of experimental parameters (the ratio of ferrous and ferric salts, types and properties of salt, pH, temperature, reaction time, and ionic strength) contributes to synthesizing IONPs with controllable shape, size, and morphology. The higher ferrous precursor concentration can cause the formation of vast quantities of seeds, which leads to increasing the yield of small nanoparticles. When the ionic strength in the system showed a growth trend, the growth and nucleation rates slowed down, promoting the emergence of nanoparticles with small size (Hui et al., 2008). Moreover, the addition of stabilizing agent can avoid the occurrence of agglomeration. Many stabilizers commonly used in modified co-precipitation methods include chelating organic anions (citrate, glucose, oleic acid, etc.) and polymer surface complexing agents (chitosan, carboxylated chitosan, starch, polyethylene glycol, etc.). Ghosh et al. have made Fe_3_O_4_ magnetic nanoparticles coated with oleic acid adopting coprecipitation (Ghosh et al., 2011). Their finding showed that conglobation could be weakened or eliminated by oleic acid, which even enhanced the efficacy of MN in killing tumor cells.

#### The Solvothermal Method

For the ideal synthesis of Fe_3_O_4_ nanoparticles, the aqueous solution or organic solvent as the reaction media is sealed in a special reactor (autoclaves) to create an environment with high temperature and high pressure. According to the type of solvent, this method is classified into a hydrothermal method and organic solvothermal synthesis. The increasing effective collisions of metal ions accelerate rapid convection of the solvent and active diffusion of solutes under solvothermal circumstances, which is conducive to the formation of nanoparticles with a narrow size distribution, uniform morphology, and better dispersion properties. Special care must be taken of the reaction factors (the types of iron source, solvent, the amount of iron source, temperature, time, etc.), exerting influence on the quality of the final product. Li et al. have fabricated nitrogen-doped graphene/Fe_3_O_4_ hybrids with enhanced microwave absorption capabilities by a facile solvothermal method (Li et al., 2017).

#### Microemulsion Route

The microemulsion route refers to the acquisition of MNPs through reactions confined to a water-in-oil or oil-in-water microemulsion stabilizing system, which could be formed by mixing surfactant, an oil phase, water phase, and co-solvent in correct proportions. The aggregation rarely occurs owing to surfactant coating on the surface of nanoparticles. The nucleation process can be developed in a controllable manner by adjusting the volume of the microemulsion. Although microemulsion approaches can be applied in the preparation of various MNPs, the final synthetic material has shortcomings of poor crystal, low magnetization, and small output due to the low reaction temperature, thus failing to meet the requirements of extensive application. Tiarks et al. have described a facile one-step synthesis of hollow polymer nanocapsules by miniemulsion polymerization of different monomers in the presence of larger amounts of hydrophobe (Tiarks et al., 2001), and their research has presented that the differences in the hydrophilicity of the oil and the polymer turned out to be the driving force for the formation of nanocapsules.

#### Sol-Gel Process

The sol-gel method is a critical means for producing materials (inorganic ceramics, glass, and nanoparticles) at room temperature by wet chemical technique (Li et al., 1991; Kawashita et al., 2000; Trewyn et al., 2007; Yu et al., 2020). This procedure involves the addition of chelator and other solvents to an aqueous solution or alkoxide containing Fe^3+^; then, stable uniform sol (nucleation) of metal oxides or metal hydroxides first occurs by hydrolysis reaction and condensation polymerization. The particles gradually grow up as the reaction proceeds, meanwhile forming a three-dimensional network gel (aging) in the liquid phase. Further heating and drying treatments are essential for the acquisition of monodisperse MNPs. Surfactants (ethylene glycol, polyethylene glycol, 1,2-propylene glycol, etc.) can modulate the nucleation and growth of the crystal nucleus, which have evident ability to optimize the process and facilitate the formation of MNPs with highly crystalline character.

Ahlawat et al. have prepared superparamagnetic NiFe_2_O_4_ nanoparticles with the average crystallite size of 9 nm by the sol-gel method (Ahlawat et al., 2011). Nearly monodispersed α-Fe_2_O_3_, γ-Fe_2_O_3_, and Fe_3_O_4_ nanoparticles were synthesized through a sol-gel approach. Moreover, more notably, the structures of iron oxide nanoparticles were switched by simply changing the drying conditions for the sol solution (Cui et al., 2013).

**FIGURE 1:**
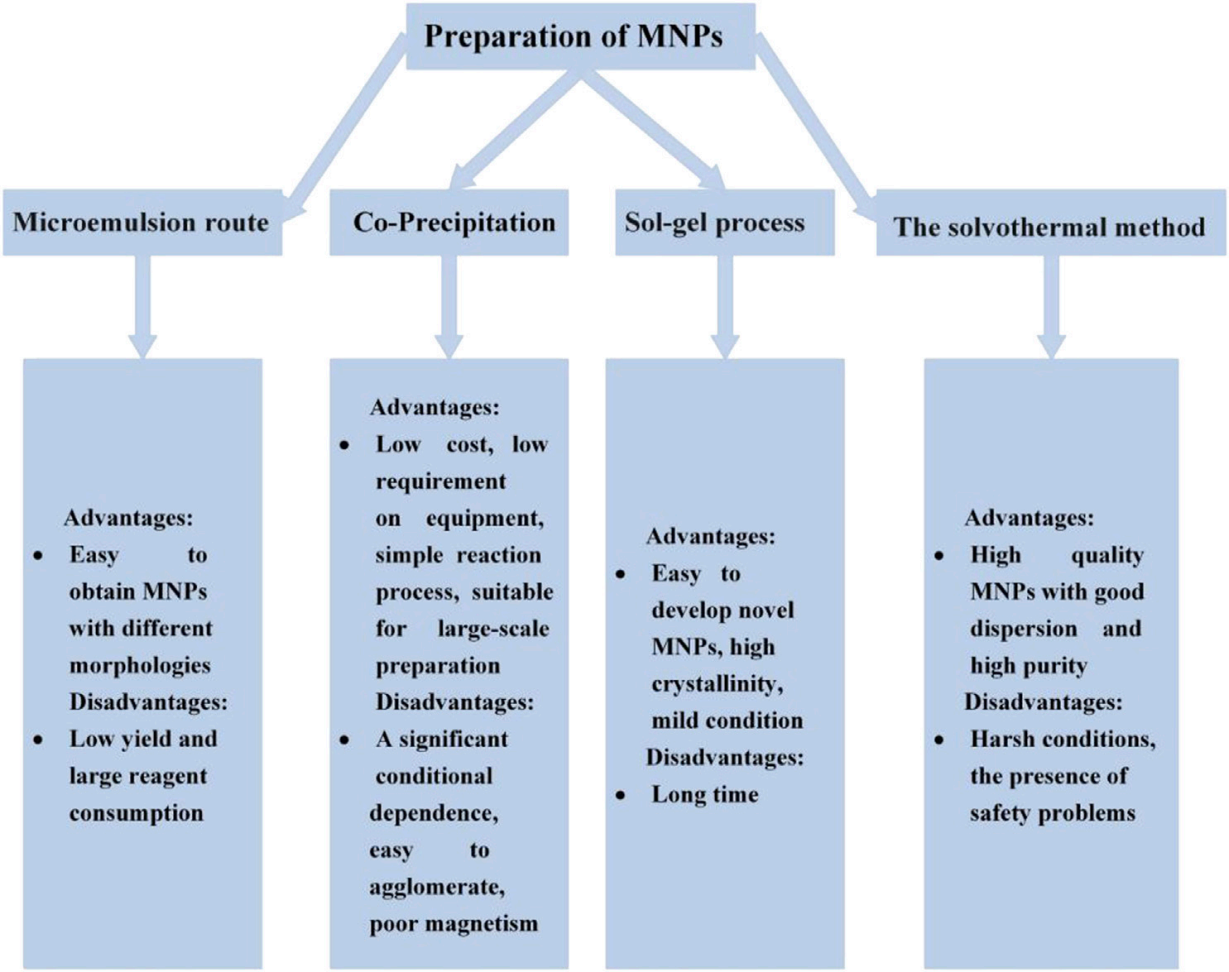
The advantages and disadvantages of the method for MNPs.

The successful preparation of MNPs is closely related to the choice of synthetic method. In order to select an appropriate preparation method, a clear understanding of the advantages and disadvantages of each method is required. Therefore, Figure 1 briefly summarizes the advantages and disadvantages of each method to provide a reference for the synthesis of MNPs.

### Surface Functionalization of MNPs

By investigating the research on MNPs in-depth, MNPs have shown great potential in nuclear magnetic resonance imaging (MRI), targeted drugs, tumor hyperthermia, drug delivery, enzyme immobilization, and protein and nucleic acid separation (Cheng et al., 2005; Hong et al., 2008; Wahajuddin and Arora, 2012). However, MNPs synthesized by traditional methods also have their shortcomings, such as high surface energy, strong anisotropic dipolar attraction, short blood circulation span, the lack of biocompatibility and dispersion stability, and poor water solubility. In addition, they are easily oxidized in the air. Several factors hinder their further development in the biomedicine field. Hence, the special modifications of the MNPs＇ surface to confer MNPs good water solubility, biocompatibility, dispersion stability, and active functional groups have become research hotspots. Usually, the purpose of a modification is accomplished by introducing a protective layer on the surface of the MNPs. The coating materials mainly include inorganic materials (silica, carbon, precious metals, etc.) and organic materials (surfactants, polymers, etc.) (Gupta and Wells, 2004; Tang et al., 2006; Wang et al., 2007; Hong et al., 2008; Huang and Hu, 2008; Zhao et al., 2008; Chertok et al., 2010; He et al., 2010). The following sections briefly introduce the strategies of MNPs modification by giving examples of relevant research works.

#### Surface-Modified Silica

The silica layer provides benefits of excellent biocompatibility, hydrophilicity and stability, reduction of the magnetic dipole effect, and easily furthered conjugation with various functional groups. The silane coupling agent will be hydrolyzed into silanol, condensing with the hydroxyl group on the MNPs surface. Moreover, the silanol group can couple with the silane reagent again to enrich the active groups, which could be bonded to certain biological molecules.

Zhang et al. have reported the controlled synthesis of single-core Fe_3_O_4_@SiO_2_ core-shell nanoparticles with a thickness of 2–20 nm via a reverse microemulsion method (Ding et al., 2012). The research has revealed the influence of different factors (the size of Fe_3_O_4_ nanoparticles and the amount of tetraethyl orthosilicate and aqueous ammonia) on the preparation of Fe_3_O_4_@SiO_2_ core-shell nanoparticles and finally presented a surface modification strategy to avoid the formation of core-free silica particles. This strategy is based on the ligand exchange between oleic acid and polyoxymethylene(5) nonylphenylether and between polyoxymethylene(5) nonylphenylether and hydrolyzed tetraethyl orthosilicate during silica coating. This approach is also suitable for silica coating of other nanometer materials, such as magnets, fluorescence, and metal nanoparticles.

#### Surface-Modified Precious Metals

To prevent magnetic core from being oxidized, noble metals are deposited on MNPs as shell materials (Fang and Zhang, 2009). Noble metals, especially Au, have extended the biomedical application of MNPs because of biocompatibility and facile biochemical modification and conjugation. Noble metals provide surface plasmon resonance (SPR) in the visible range and amplify the Raman signal of the adsorbed molecules (Wang et al., 2016; Izadiyan et al., 2019).

Nagarjuna et al. have shown a solid-state synthetic approach (Solvent-less synthesis) for the preparation of Fe_3_O_4_@M (where M = Au, Ag, and Au-Ag alloy) core-shell nanostructures (Nalluri et al., 2019). This route comprises a physical grinding of a metal precursor and magnetite core to realize a uniform coating of the precursor over the Fe_3_O_4_ core, followed by calcination. The prepared Fe_3_O_4_@Ag and Ag-rich alloy catalysts have exhibited high catalytic efficacy for hydrogen generation than Fe_3_O_4_@Au and Au-rich alloy synthesized in this study.

Tang et al. have reported a simple chemical approach for the fabrication of Fe_3_O_4_@Ag nanoparticles and later constructed advanced immunosensors based on core-shell Fe_3_O_4_ @Ag magnetic nanoparticles, showing an excellent electrochemical response selective to the carcinoembryonic antigen (Tang et al., 2006).

Liu et al. have used NaBH_4_ as a reductant to reduce Pt^4+^ onto the surfaces of Fe_3_O_4_-core nanoparticles for the formation of Pt shells; finally, translucent and homogeneous Fe_3_O_4_@Pt nanocomposites were produced (Liu et al., 2013). Meanwhile, the electrochemical differences between Fe_3_O_4_@Pt core-shell nanocomposites and nanoparticles (Fe_3_O_4_ nanoparticles and Pt nanoparticles) with a single component and simple structures were investigated in this research. Pt shells could offer more electrocatalytic activity, while magnetic Fe_3_O_4_ core could give benefits of larger surface area.

Lin et al. have synthesized Fe_3_O_4_@Au core-shell nanoparticles and coated streptavidin on their surfaces (Lin et al., 2019). The streptavidin-coated Fe_3_O_4_@Au nanoparticles possessed the enhanced magneto-optical Faraday effect due to the SPR of the gold shell. The process involves the fabrication of Fe_3_O_4_ MNPs by co-precipitation, the reduction of Au^3+^ to form Fe_3_O_4_@Au core-shell MNPs using hydroxylamine hydrochloride (NH_2_OHHCl), and the linkage of streptavidin onto the gold surface by 11-mercaptoundecanoic acid to supply a carboxyl group for bioconjugation subsequently.

#### Surface-Modified Carbon

The carbon layer can build a protective barrier against oxidation and acid erosion, so carbon-modified MNPs possess characteristics of high chemical and thermal stability. Moreover, as an essential type of absorbent, carbon-coated nanomaterials, have a bright future in water purification for the removal of contaminants because of their large specific surface area, high separation properties, and easy functional modifications (Zhang and Kong, 2011; Zhou et al., 2017).

**FIGURE 2:**
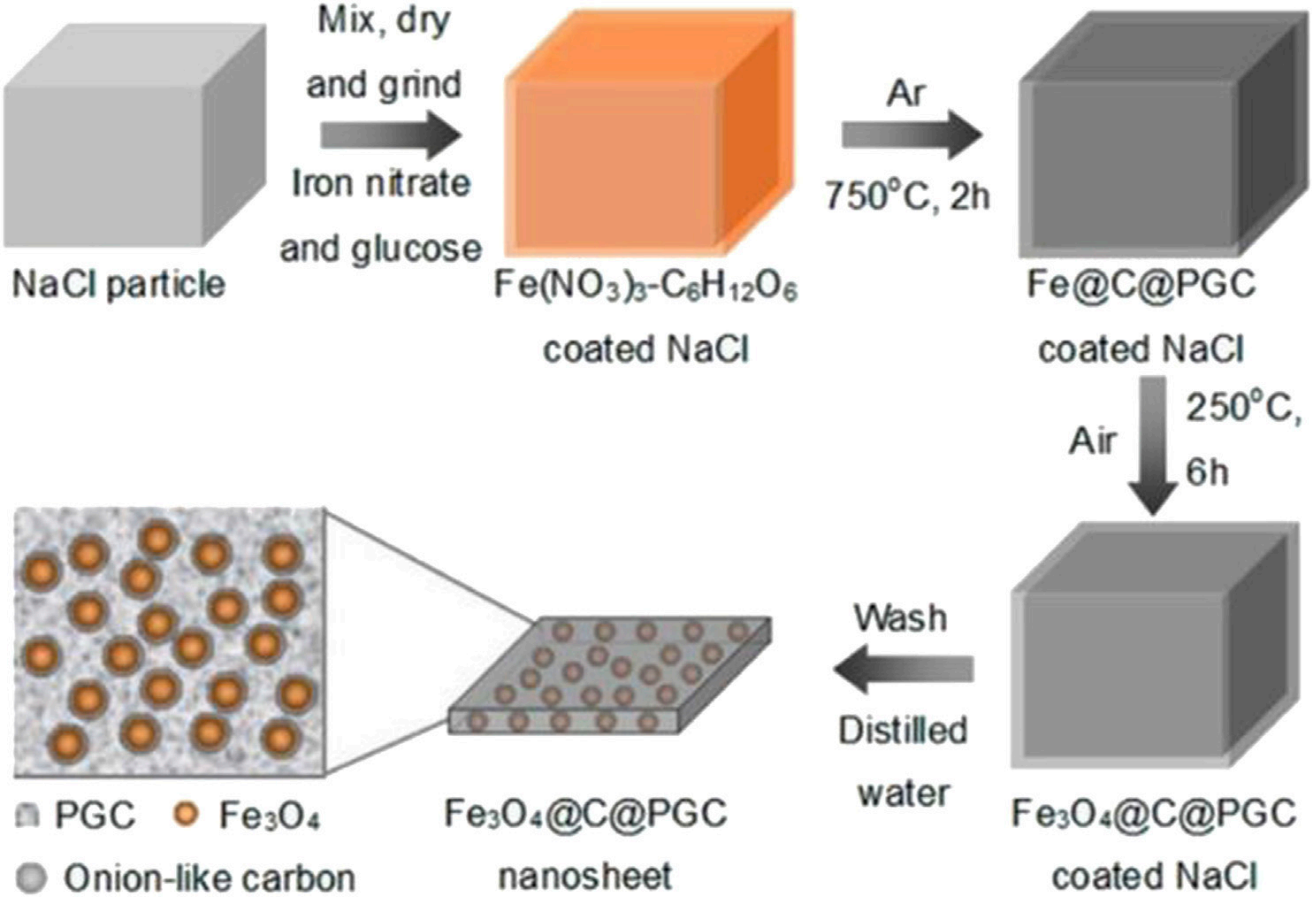
Schematic illustration of the *in situ* technique to fabricate 2D Fe_3_O_4_@C@PGC nanosheets by using the surface of NaCl particles as the template. Reprinted with permission from (He et al., 2013). Copyright (2021) American Chemical Society.

He et al. have pointed out the successful synthesis of novel carbon-encapsulated Fe_3_O_4_ nanoparticles embedded in 2D porous graphitic carbon nanosheets by a facile *in situ* synthesis method with the assistance of water-soluble NaCl particles as Figure 2 (He et al., 2013). The thin onion-like carbon shells can effectively avoid exposing Fe_3_O_4_ to the electrolyte and maintain the structural stabilization of Fe_3_O_4_ nanoparticles. The conductive 2D porous graphitic carbon nanosheets can inhibit the aggregation of Fe_3_O_4_ nanoparticles. The electrode based on this material is expected to be applied in adsorbents, catalysts, and sensors in many scientific domains.

Lu et al. have prepared yolk-shell nanostructured Fe_3_O_4_@C nanoparticles by utilizing a facile one-step process and selective chemical etching (Lu et al., 2017). The novel structures gave them enhanced intrinsic peroxidase mimetic activity and excellent superparamagnetic properties.

Zeng et al. have reported a new kind of monodisperse magnetic rattle-type nanocapsule with a C_18_ -modified interior cavity and a functional double-layered shell, which could effectively extract trace organic targets from water samples (Zeng et al., 2013).

#### Surface-Modified Polymers

Candidate polymers for surface modifiers can be either natural polymers or synthetic polymers. Dextran, chitosan, starch, polyethylene glycol (PEG), polyvinyl alcohol (PVA), polyethyleneimine (PEI), polyvinylpyrrolidone (PVP), polydopamine (PDA), and polyamidoamine dendrimers (PADs) are some universally researched and applied polymers for MNPs modification.

Chitosan is the N-deacetylation product of chitin with prominent features such as hydrophilicity, biocompatibility, antibacterial properties, and significant affinity for many biomacromolecules (Chang and Chen, 2005). Researchers have found that chitosan can absorb heavy metal ions, which attributes to the presence of abundant amine groups (Yuwei and Jianlong, 2011). Li et al. have employed chitosan as an effective stabilized agent for producing Fe_3_O_4_- chitosan nanoparticles with magnetic cores and polymeric shell (Li et al., 2008). Firstly, 23 nm Fe_3_O_4_ nanoparticles were prepared by hydrothermal method using H_2_O_2_ as an oxidizer; afterward, chitosan and Fe_3_O_4_ aqueous slurry were mixed in suitable proportion using reverse-phase suspension crosslinking method to make the MNPs with an amine group. The saturated magnetization of composite nanoparticles could reach 21.5 emu g ^−1^. Cao et al. have prepared magnetic Fe_3_O_4_/chitosan nanoparticles with quasi-spherical or ellipsoidal morphology by a simple reduction–precipitation method consisting of reducing Fe^3+^ with sodium sulfite, precipitation with ammonia at room temperature, and then crosslinking with epoxy chloropropane (Cao C. et al., 2014). Brilliant red X-3B was selected as a dye pollutant to evaluate the adsorption behavior of magnetic Fe_3_O_4_/chitosan nanoparticles by batch adsorption experiments. This finding revealed that the adsorption performance of magnetic Fe_3_O_4_/chitosan nanoparticles was remarkably dependent on initial pH and dosage of the adsorbent.

**FIGURE 3:**
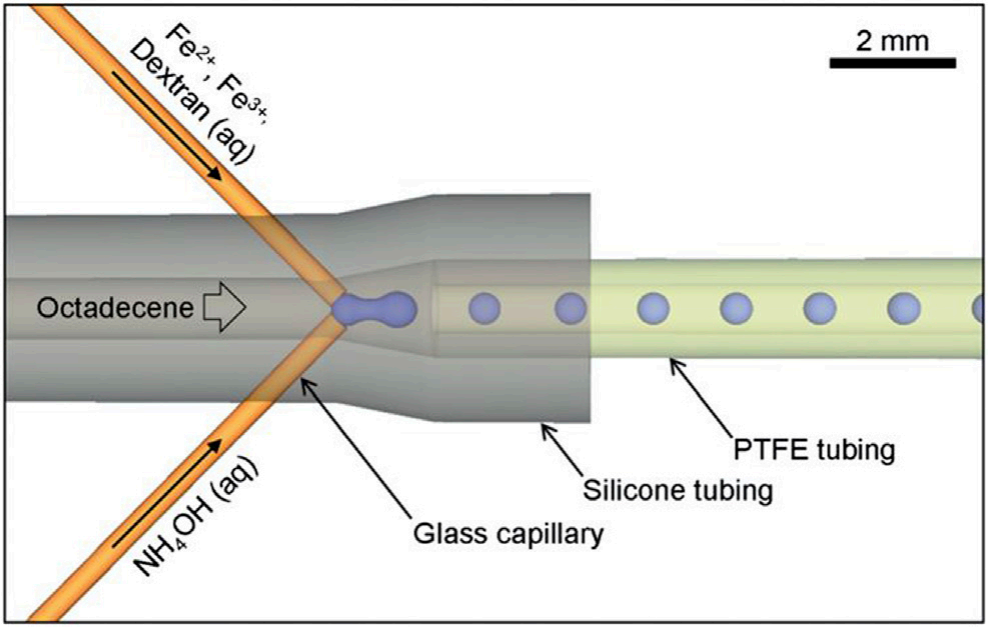
Schematic of the capillary-based droplet reactor showing the injection of separate precursor streams of Fe^2+^/Fe^3+^/dextran and NH_4_OH into a continuous stream of octadecene carrier fluid; iron oxide nanoparticles are formed inside the droplets. Reprinted with permission from (Kumar et al., 2012). Copyright (2021) Royal Society of Chemistry.

Dextran as a natural polysaccharide showed powerful advantages, such as biocompatibility, biodegradability, water solubility, chemical inertness in biological environments, low toxicity to cells, and prolonged blood circulation time (Saraswathy et al., 2014). Dextran can also stabilize MNPs by triggering interparticle repulsion, which reduces magnetic attraction between particles (Khot et al., 2013). Unterweger et al. have prepared dextran-coated superparamagnetic iron oxide nanoparticles with five different sizes using the cold gelation method and investigated the size dependency of their imaging properties (Unterweger et al., 2018). The synthesized nanoparticles displayed no irritation potential and exerted no negative effects on vessels *in vivo*. Peng et al. have developed dextran-coated superparamagnetic nanoparticles as magnetic carriers for the delivery of doxorubicin (Peng et al., 2015). This conjugate is of high efficiency with pH-dependent drug release and exhibits a desirable anti-tumor activity with lower cytotoxicity. Kumar et al. have reported a novel approach using a stable passively driven capillary-based droplet reactor as Figure 3 to prepare dextran-coated superparamagnetic iron oxide nanoparticles with a narrow size distribution, favorable stability, and high crystallinity (Kumar et al., 2012).

PEG, a common encapsulating material for the stabilization of magnetic iron oxide nanoparticles (MIONPs), can extend the circulation times of MNPs *in vivo* and increase the hydrodynamic diameter of particles (Cole et al., 2011; Larsen et al., 2012). Fe_3_O_4_ nanoparticles coated with PEG have fine biocompatibility, longer blood circulation time, and high cell internalization efficiency. Zhao et al. have produced Fe_3_O_4_/PEG core-shell nanoparticles with a diameter of 10–40 nm by two-step additions of oleate sodium and PEG, respectively (Zhao et al., 2010). Exposed in the alternating current magnetic field for 100 s, the temperatures of physiological saline suspensions containing Fe_3_O_4_/PEG nanoparticles are 72.2°C. Thus, this material can be applied in localized hyperthermia treatment of cancers as thermoseeds. GUPTA et al. have modified the surfaces of the superparamagnetic nanoparticles using PEG (Gupta and Curtis, 2004). The PEG-coated nanoparticles exerted no influence on the cell adhesion behavior, morphology, and their internalization into endosomes, which made it possible to label a variety of cells with high efficiency. Nazli et al. have reported the surface modification of MIONPs with covalently crosslinked biofunctional PEG hydrogel (Nazli et al., 2012). The material obtained showed outstanding stability, viability, and specific cellular uptake. PEG hydrogel-coated MIONPs were further functionalized with the fibronectin-derived arginine-glycine-aspartic acid-serine (RGDS) sequence to acquire a biofunctional PEG hydrogel layer around the nanoparticles. RGDS-bound PEG hydrogel-coated MIONPs showed a 17-fold higher uptake by the human cervical cancer HeLa cell line than that of amine-coated MIONPs. Furthermore, this novel finding allows for the coating of MIONPs by nano-thin hydrogel layers that may prevent the adhesion of undesirable cells and proteins. It may be a promising technique for cellular uptake in target tissues in a specific manner.

**FIGURE 4:**
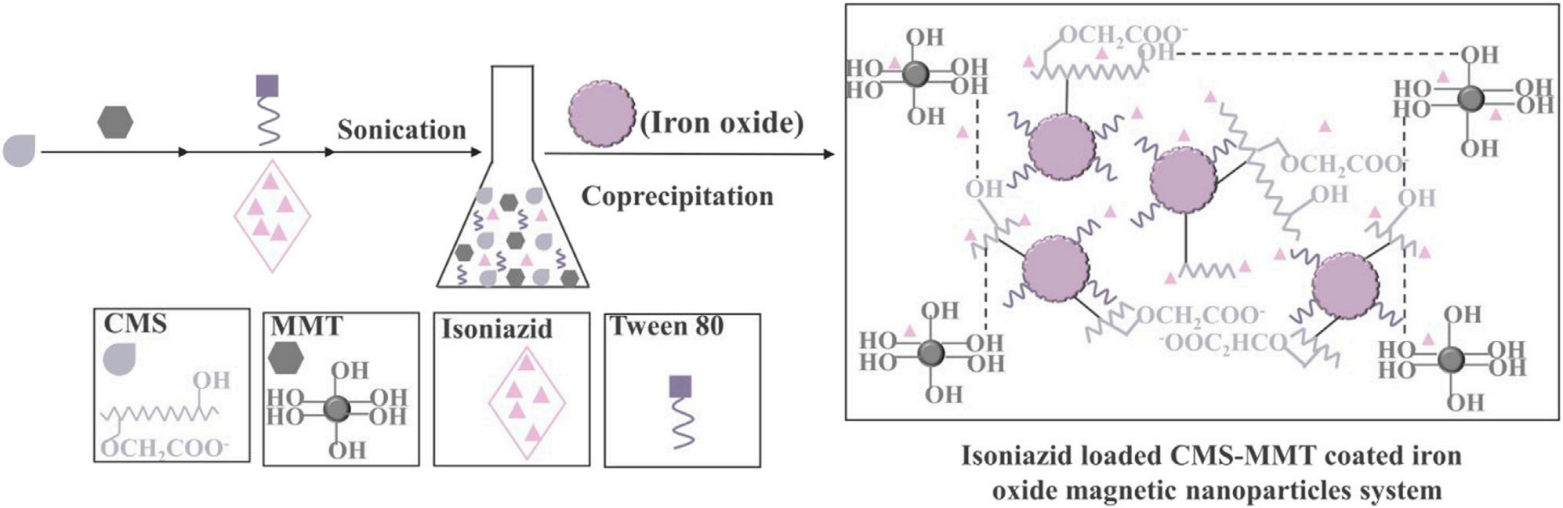
Schematic representation of the preparation of isoniazid-loaded CMS-coated iron oxide/MMT nanoparticles (Saikia et al., 2015).

Starch, which consists of amylose and amylopectin, is a popular coating material for the purpose of improving MNPs properties due to its evident properties of hydrophilia, biocompatibility, biodegradability, low costs, ready availability, and non-toxicity (Kim et al., 2009; Rafiee and Khodayari, 2015; Andjelković et al., 2018). Mahmoud et al. have made excellent use of iron oxide waste byproducts from the steel industry to fabricate Fe_2_O_3_ nanoparticles-starch nanocomposite using starch as a good stabilizer and formaldehyde as a biodegradable polymer (Mahmoud et al., 2019). This nanocomposite showed a strong affinity to extract Pb (II), Hg (II), and Cd (II) due to the excellent affinity of interacting with loaded oxygen donor atoms of the hydroxyl groups. The result displayed that the designed nanocomposite was highly selective for Pb (II) as the highest extraction efficiency values achieved were 98, 97, and 98% from tap water, seawater, and industrial wastewater, respectively. Saikia et al. have used carboxymethyl starch as an efficient drug carrier to modify MNPs as Figure 4, which was an important modified starch owing to the presence of negatively charged groups (CH_2_COO^−^) (Saikia et al., 2015). Moreover, the higher amount of surfactants was beneficial to the generation of smaller nanoparticles. This material exhibited better swelling and drug release at pH 7.4.

Except for the polymers emphasized above, other polymers also ameliorate the comprehensive performance of MNPs and broaden their application scope. Fang et al. have reported the successful production of multilayered coating single magnetic nanoparticles functionalized by PDA and Schiff base ligand (3-aminopyridine-2-carboxaldehyde N(4)-methylthiosemicarbazone, HL) (Fang et al., 2019). The nanoparticles showed the aggregation-induced emission effect by embedding In^3+^ into their nanostructure, which can be used as a fluorescent detection and therapeutic method. Sahin et al. have activated PVA-coated MNPs with glutaraldehyde for covalent immobilization of trypsin (Sahin and Ozmen, 2020). The final prepared material had a high protein loading capacity (79%) and activity recovery (99%) in the optimum immobilization conditions. Similarly, immobilized trypsin on this nanocomposite exhibited higher operational stability and higher thermal and storage stability. The coating of PVA with hydroxide on the surface of MNPs could protect against oxidation and functionalize enzyme immobilization. Khoobi et al. have modified MNPs using PEI, which could act to stabilize nanoparticles as a capping and reducing agent (Khoobi et al., 2015). The formation of PEI-functionalized MNPs was dependent on electrostatic adsorption of positively charged PEI on the negatively charged surface of MNPs. Meanwhile, they have reported that PEI grafted on silica-coated Fe_3_O_4_ nanoparticles could be novel catalyst alternatives for the one-pot synthesis of pyrano[3,2-c] chromene, chromenes, and spiro-oxindoles derivative because they avoided the use of large volumes of hazardous organic solvents and simplified experimental and work-up procedures. Huang et al. have investigated the influence of PVP-coated iron oxide nanoparticles with different sizes on cellular uptake and MRI (Huang et al., 2010). The fabrication of PVP-coated iron oxide nanoparticles with different particle sizes was achieved by controlling the ratio of PVP to iron carbonyl (Fe (CO) _5_). The decrease in particle sizes was observed with the increase of PVP concentration. PVP immobilized on the surface of the IONPs by coordination interaction of carbonyl group resulted in good dispersion of the IONPs in water and controlled growth of IONPs. Besides, MRI contrast enhancement of PVP-coated iron oxide nanoparticles within the liver is highly size-dependent. This research provided optimized conditions for engineering nanoparticles to be applied in biomedical imaging. The high density of surface terminal groups, good structural homogeneity, and controllable size of PADs were great for extracting heavy metals. The study of Maleki and coworkers has designed new second-generation PADs functionalized magnetic nanoparticles (Fe_3_O_4_@G_2_-PAD) (Maleki et al., 2019). Fe_3_O_4_@G_2_-PAD suspension was dropped on the smoothed magnetic carbon paste electrode (MCPE) to fabricate Fe_3_O_4_@G_2_-PAD/MCPE for multi-element detection. The modified MCPE displayed excellent electrochemical properties for the detection of Pb^2+^ and Cd^2+^ (obtained low detection limits: 0.17 ng/ml for Pb and 0.21 ng/ml for Cd), which could better meet the threshold limit (10 ng/ml for Pb and 3 ng/ml for Cd) established by the World Health Organization in drinking water. Ma et al. have prepared PAD dendrimers functionalized magnetic graphene oxide by step-by-step growth chemical grafting approach for the adsorption of Hg (II) in aqueous solution (Ma et al., 2017). The synthesized material possessed good absorption performance for Hg (II) with the maximum adsorption capacity of 113.71 mg/g, and this finding has revealed that the adsorption process was mainly a monolayer chemical adsorption.

### Advanced Polymerization Technique for Magnetic Molecularly Imprinted Polymers

The conventional approaches used to fabricate MMIPs are mostly embedding methods, mainly including bulk polymerization, suspension polymerization, emulsion polymerization, and precipitation polymerization. The aforementioned methods are more or less limited in their feasibility and practical application due to specific reasons. For example, the imprinting sites located inside the polymer are inhomogeneous, resulting in the time-consuming, laborious, and tedious elution process of template molecules. Additionally, the elution process inevitably destroys the effective imprinting holes; the introduction of dispersant and surfactant interferes with the interaction between the template molecule and the functional monomer, potentially influencing the adsorption specificity of MMIPs; the distribution of obtained product size is within a wide range. As an emerging technology, surface molecular imprinting technology can disperse most of the recognition sites on the surface of MMIPs. Compared with the traditional embedding methods, the prepared particles have an even distribution in size; the accessibility of the recognition cavity is beneficial to improve the recognition efficiency of the target molecule. Various advantages broaden the application scope of surface imprinting technology. As follows, we try to present novel examples for the discussions of each synthetic pathway for surface molecularly imprinted polymers.

#### Pickering Emulsion Polymerization

MIT based on Pickering emulsion is a new type of MIT developed on the traditional emulsion MIT. An emulsion can be formed by uniformly dispersing an aqueous solution in another immiscible solution. This dispersion system usually requires the addition of surfactants to raise its stability. Surfactants always contain non-polar, lipophilic hydrocarbon chain parts and polar hydrophilic groups. The formation of oil-in-water (O/W) or water-in-oil (W/O) emulsion is achieved by adding surfactants to the emulsion. The resulting emulsion, usually called Pickering emulsion, replaces environmentally harmful surfactants with solid particles. The type of Pickering emulsion (O/W, W/O) is determined by the contact angle θ between particles and aqueous solution, measuring the wettability of the solid particles. The contact angles θ below and over 90°, respectively, correspond to O/W and W/O emulsion. In contrast, solid particles are more stable at the interface of oil and water (Zhu et al., 2016). This technique establishes the recognition cavity on the polymer surface, thereby boosting the adsorption efficiency; well realizes the preparation of molecularly imprinted polymers with specific recognition in the aqueous solution; adopts solid particles with low toxicity, good stability, and easy modification as a stabilizer, therefore effectively overcoming the defects of surfactants.

**FIGURE 5:**
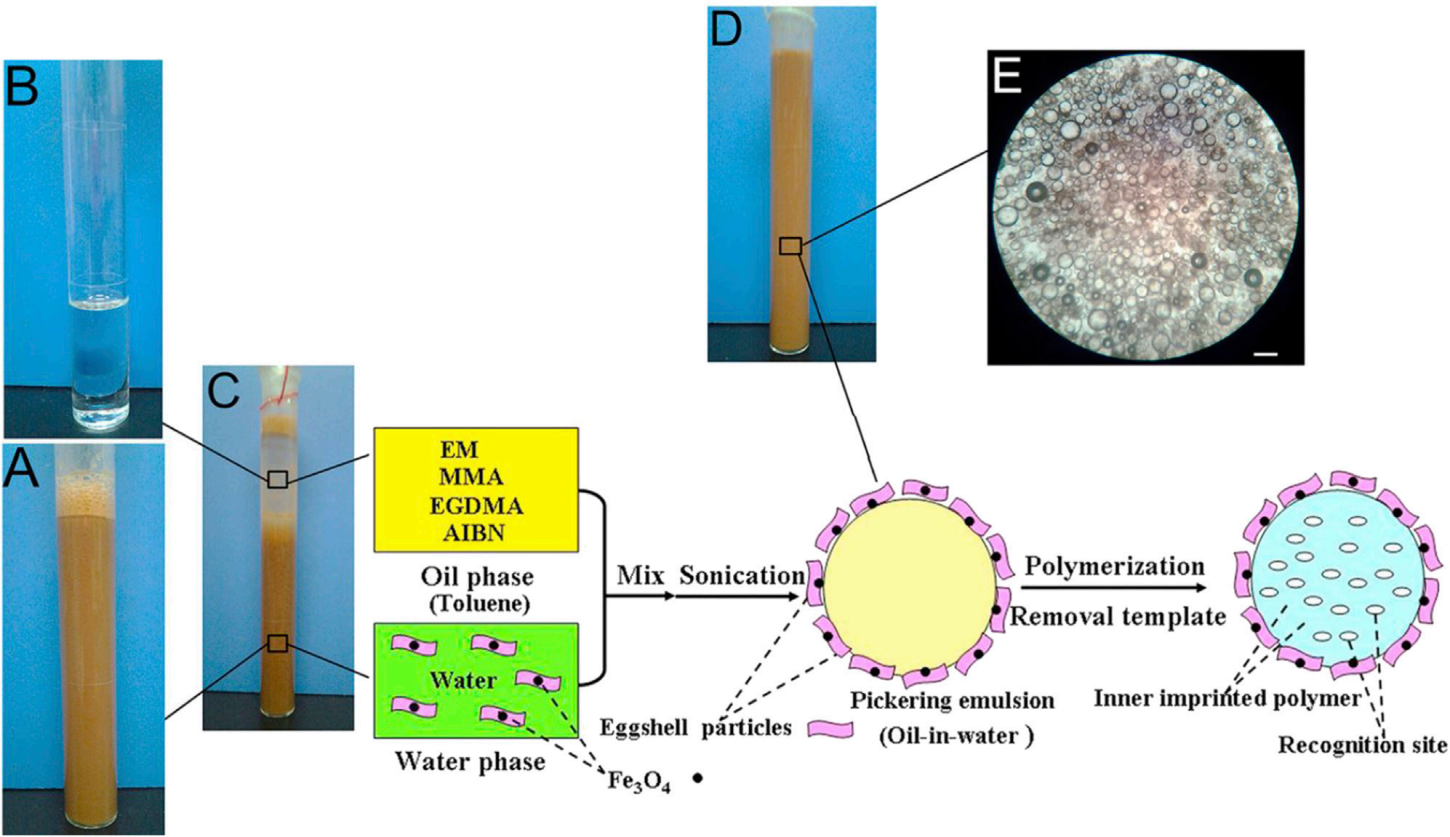
Synthesis approach of MMIPs by Pickering emulsion polymerization: Photographs of a water-phase dispersion **(A)**, an oil-phase dispersion **(B)**, and a Pickering emulsion **(C)** before and **(D)** after ultrasonification and the micrographs of emulsion droplets stabilized by eggshell particles **(E)** (scale bars: 100 μm). Reprinted with permission from (Zhu et al., 2016). Copyright (2021) Elsevier.

Zhu et al. have obtained MMIPs by magnetic eggshells-stabilized Pickering emulsion imprinting polymerization (Zhu et al., 2016). The eggshell is a natural material consisting of inorganic-organic components such as calcium carbonate, calcium phosphate, organic matter, and magnesium carbonate. Eggshell can be chosen as a stabilizer for Pickering emulsion because the main component of eggshell is extremely hydrophilic, while a handful of organic ingredients can reduce hydrophilicity and enhance wettability. Meanwhile, eggshell particles can bond closely with Fe_3_O_4_ particles as magnetic carriers due to their strong adsorption capacity. As shown in Figure 5, toluene and water constitute an emulsion. The eggshell particles and Fe_3_O_4_ nanoparticles were dispersed in the double distilled water to form the water phase. The sole functional monomers (methyl methacrylate, MMA), cross-linkers (ethylene glycol dimethacrylate, EGDMA), initiator (2,2-azobis (2-methylpropionitrile), AIBN), and the template molecule (erythromycin, EM) were dissolved in toluene, which was used as the oil phase. A stable Pickering emulsion was prepared with a digital sonicator.

#### Living/Controlled Radical Polymerization

Like any chain polymerization, radical polymerization consists of four reactions: initiation, propagation, transfer, and termination (Braunecker and Matyjaszewski, 2007). Conventional free radical polymerization failed to synthesize polymers with controllable molecular weight and polydispersity due to slow initiation, fast chain interactions, and undesirable chain termination, which could result in heterogeneous architectures and low selectivity (Dai et al., 2012). The concept of CRP was first proposed by Szwarc et al. and usually refers to a polymerization without chain termination and irreversible chain transfer (Szwarc et al., 1956). With the further study of CRP, Quirk and Lee have evaluated the typical feature of CRP and modified its definition as polymerization with reversible termination and reversible chain transfer (Quirk and Lee, 1992). CRP has several characteristics that are clearly different from traditional free radical polymerization as follows: the chain initiation rate is far greater than the chain growth reaction rate, which leads to the narrow molecular weight distribution and controllable chain structure; the lifetime of growing chains is extended; the proportion of dead chains is usually small (＜10%) in CRP; the termination rate reduces with time, which makes the growth of polymer chains controllable (Braunecker and Matyjaszewski, 2007). There are generally two methods for CRP: atom transfer radical polymerization (ATRP) and reversible addition-fragmentation chain transfer (RAFT).

ATRP is a promising surface imprinting technique due to its ability to graft polymer on carriers, having more applicable monomer, moderate reaction conditions, and the tolerance of high polar solvents (Li et al., 2012). ATRP transfers halogen atoms from initiator to monomer and finally continuously transfers them to the growing polymer chain under the catalysis of transition metal complexes to achieve polymerization. Li et al. have firstly coated the chitosan layer containing γ-Fe_2_O_3_ nanoparticles onto the surface of the yeast to form the magnetic yeast composites (mag–yeast) and then obtained elliptical MMIPs for the recognition of cefalexin by ATRP in a mixture of methanol and deionized water (Li et al., 2012). This environmental approach dramatically shortened the polymerization time, and the prepared MMIPs have adsorptive capacity. Mao et al. have prepared Fe_3_O_4_ nanoparticles modified with dopamine to introduce amine groups (Mao et al., 2015). Meanwhile, the PDA layer was coated on the Fe_3_O_4_ nanoparticles in 10 mM Tris–HCl buffer solution (pH 8.5); then, the initiator (2-bromoisobutyryl bromide) was grafted on the surface of PDA. Finally, the MIP layer was formed on the surface of Fe_3_O_4_ by copolymerization, using an organometallic catalyst comprising Cu(I)Br, sulfamethazine (SMZ) as a template, methacrylic acid as a functional monomer, and ethylene glycol dimethacrylate as a crosslinking agent. This work shows that the controllable characteristic of ATRP is conducive to the growth of a uniform MIP layer with adjustable thickness, providing a large adsorption capacity, fast kinetics about 40 min to equilibrium, and a considerably high imprinting factor. Dai et al. have proposed an innovative method for obtaining MMIPs via atom transfer radical emulsion polymerization (Dai et al., 2012). This study successfully applied ATRP in an emulsion system to prepare the MIPs, which combined the advantages of ATRP and emulsion polymerization, supplying both the homogeneous structures and nanoparticles. The prepared MMIPs exhibited good features such as excellent specificity, adsorption ability, and thermal stability using this technique. Cao et al. have synthesized MMIPs using magnetic particles as a core through reverse atom transfer radical precipitation polymerization at the surface of MNPs (Cao Z. et al., 2014). The as-prepared MMIPs could selectively recognize and rapidly remove SMZ molecules from an aqueous medium as nano-absorbents with a 15 nm uniform imprinted shell.

RAFT polymerization is arguably an extensively used approach for the synthesis of polymers with specific structures and multifunctional properties. In the RAFT polymerization reaction, the dormant species ([S=C(S)-Z]) are transferred between the active and the dormant chain, realizing the dynamic reversible equilibrium between the active free radical chain and the “dormant species,” which can effectively maintain the activity of the polymerization reaction and offer the obtained polymer a controllable molecular weight and narrow polydispersity (Chiefari et al., 1998). Shao et al. have firstly employed distillation-precipitation to prepare Fe_3_O_4_ nanoparticles modified by 4-vinylbenyl chloride, which easily introduced azide groups on the surface of MNPs to form the relatively large amount of benzyl chloride groups (Shao et al., 2017). Afterward, the alkyne-terminated RAFT chain transfer agent was then immobilized onto the surface of Fe_3_O_4_ nanoparticles with high efficiency. Finally, the highly uniform imprinted thin film was fabricated on the surface of RAFT agent–modified Fe_3_O_4_ nanoparticles. The prepared products showed exceptional molecular imprinting effects, fast rebinding kinetics, and excellent selectivity to compounds with similar configurations. Li et al. have synthesized selective MMIPs for the quantitative determination of 17 β-estradiol (Li et al., 2011). The synthesis of the “three-in-one” beads via a multistep procedure mainly involves preparation of Fe_3_O_4_ nanoparticles, silica-shell deposition, attachment of fluorescein isothiocyanate onto the silica surface, silica-shell deposition again, MIP-functionalization onto the silica surface, and final extraction of 17 β-estradiol and generation of the recognition site. The synthesized multifunctional ‘‘three-in-one’’ beads combine magnetism, excellent fluorescence, and higher selectivity characteristics. This material not only selectively detects nonfluorescent estrogenic disrupting chemicals without the need for any inducers and derivatization but also improves the separation behavior and selectivity of molecular imprinting.

### Advanced Imprinting Technique

#### Dummy Imprinting Technique

In order to effectively tackle a series of problems such as template leakage, the target template molecule with high costs, high toxicity, unsafety for operation, low solubility, fragility, or degradability, dummy imprinting technology has begun to attract the researchers’ attention. In short, the structural analog of the target template or the part similar to the target template is used as a pseudo-template to synthesize MIPs. Wang et al. have successfully synthesized molecularly imprinted silica employing 1,10-phenanthroline-4-carboxylic acid as the dummy template, a structural analog of aristolochic acid I, reasonably settling the problems of the template (aristolochic acid I), such as being toxic, expensive, and unstable and template leakage (Wang et al., 2020).

#### Double Imprinting Technique

MIPs based on single-template imprinting technology often only display good recognition ability for target templates. In contrast, the recognition effect of their structural analogs is not optimal, limiting their application in the identification of multi-target molecules in complex environment samples. Multi-template imprinted polymers often have two or more imprinting cavities, which can well satisfy the recognition, extraction, and separation of structurally related homologs or analogs (Chen et al., 2019). Researchers have also confirmed that double-template imprinted polymers possessed higher adsorption capacity, higher selectivity, and better imprinting performance than MIPs prepared by single-template imprinting technology (Meng et al., 2009). Wei et al. have fabricated a magnetic mesoporous dual-template molecularly imprinted polymer with a specific recognition capability, using chloramphenicol and florfenicol as dual-template molecules, α-methacrylic acid and Fe_3_O_4_@mSiO_2_@-CH = CH_2_ as dual functional monomers, and ethylene glycol dimethyl methacrylate as a crosslinking agent (Wei et al., 2016).

## Magnetic Molecularly Imprinted Polymers in Antibiotic Detection: Clean-Up Procedure

Antibiotics are defined as a class of drugs produced by certain microorganisms, fungi, and bacteria in low concentrations, inhibiting the growth of bacteria, fungi, and parasites (Samanidou and Nisyriou, 2008; Kummerer, 2009; Moreno-Bondi et al., 2009). Antibiotics are developed for clinical therapy due to their capacity to fight microbial populations. The vital function of antibiotics in the field of biomedicine cannot be neglected. However, the problem of antibiotic residue caused by the irregular use of antibiotics also needs our attention. The potential harm of antibiotic residues to animals, humans, and the environment has raised great concerns. Antibiotic residues will induce the generation of antibiotic-resistant bacteria, serving as reservoirs of antibiotic-resistant genes. Through horizontal transfer, the resistance genes may be transferred to human pathogens, which will eventually lead to a human health crisis, including the allergic reactions of sensitive individuals and the failure of treatment-related infections (Heuer et al., 2009).

Currently, many countries set acceptable levels of tolerance to these antibiotics. In 1990, the European Commission sat down a series of procedures to establish maximum residue limits in animal foodstuffs. Maximum residue limits can be defined as the maximum concentration of labeled residues (e.g., parent compound and metabolite) caused by antibiotic use (Mitchell et al., 1998).

The development of various antibiotic determination methods is a key means for controlling antibiotic resistance. At present, the common approaches of antibiotic detection mainly contain biosensors, immunochemical techniques, and chromatographic methods (Pastor-Navarro et al., 2004; Himmelsbach and Buchberger, 2005; Kantiani et al., 2009; Raz et al., 2009; Kim et al., 2010; Wang et al., 2014; Luo et al., 2015). A series of potentially fatal problems, such as trace antibiotic residues in the environment, matrix interference, and the presence of co-eluting components, not only hinder qualitative and quantitative analysis of analytes but also influence the performance of the aforementioned methods for the detection of antibiotics (Regal et al., 2012). In addition, the identification of antibiotics may be pH-dependent for immunochemical techniques, such as antibodies with a wide recognition profile of fluoroquinolone antibiotics synthesized by Pinacho et al. (2012); the detectability of this material for fluoroquinolones (FQs) decreased when the pH values are around 7.5. Hence, effective sample pretreatment to eliminate potential interference and enrich target antibiotics is important for subsequent antibiotic determination. MMIPs are just the right way to solve these problems. The advantages of MMIPs as selective solid-phase extraction sorbent in the analysis of antibiotic residues have been widely confirmed by researchers. In the following section, we will briefly discuss novel MMIPs in the pretreatment steps of antibiotic detection by introducing the innovative work of researchers.

There are numerous fresh materials as support carriers to improve the performance of MMIPs. Carbon nanotubes (CNTs) can be utilized to synthesize MMIPs because of their excellent adsorption ability due to their extremely large surface area and structural characteristics (Chen et al., 2015; Zhao et al., 2015). Chen et al. have synthesized a novel quinolones MIP on the surface of ethylenediamine-functionalized magnetic carbon nanotubes (EDA@Mag-CNTs-MIP) by a simple reaction starting from carboxyl-CNTs (Chen et al., 2015). The following are the main steps: 1) synthesis of EDA@Mag-CNTs nanoparticles by solvothermal method; 2) thermally initiated polymerization of functional monomer (glycidyl methacrylate), crosslinker (divinylbenzene) in the presence of an initiator and dispersing medium, and grafting reaction between EDA in the EDA@Mag-CNTs nanoparticles and epoxyl groups in co-poly (glycidyl methacrylate-divinylbenzene); 3) elution of template molecules. This research reflects several benefits of EDA@Mag-CNTs as a promising support carrier of MIPs, including a large surface area, high saturation magnetization, mechanical strength, and hydrogen bonding between -O−/−F/-COOH in template molecule and -NH- in EDA@Mag-CNTs-MIP. In addition, with using EDA@Mag-CNTs-MIP as the adsorbent, the combination of magnetic molecularly imprinted polymer matrix solid-phase dispersion extraction procedure with ultra-fast liquid chromatography-tandem quadrupole mass spectrometry was developed to determine FQs in river water; EDA@Mag-CNTs-MIP presented the higher extraction capacity with recoveries between 80.2 and 116%. The limits of quantification for the FQs were between 0.26 and 1.78 ng/L. Zhao et al. have fabricated the core-shell MMIPs on the surface of magnetic CNTs to detect sulfamethoxazole (Zhao et al., 2015). The resulting MMIPs exhibited a highly improved imprinting effect, fast adsorption kinetics, and high adsorption capacity and could be applied to rapidly extract sulfamethoxazole in the milk and honey samples. Xiao et al. prepared MMIPs on the surface of CNTs, which achieved a high selectivity toward FQs (Xiao et al., 2013). The excellent mechanical properties of encapsulated CNTs in MIPs make the resulting materials have more imprinted holes, which improves the separation performance of the synthesized materials for antibiotics.

Halloysite nanotubes (HNTs) are an obvious hollow tubular structure with the outer surface and the inner surface consists of a tetrahedral (Si-O) and an octahedral (Al-OH) sheet, attracting great interest in sample separation owing to superior characteristics including high porosity, large surface area, and tunable surface chemistry (Yuan et al., 2015; Ma et al., 2016). Inspired by biomimetic *Setaria viridis*-like structure, Ma et al. have provided a new idea for the preparation of hydrophilic magnetic surface molecularly imprinted core-shell nanorods (HMMINs) with magnetic halloysite nanotubes (HNTs) as nano-cores, via a two-step surface-initiated atom transfer radical polymerization in a green alcohol/water mixture solvent at room temperature (Ma et al., 2016). HMMINs showed a well-defined core-shell structure with ultrathin imprinted film (12 nm) and hydrophilic polymer brushes (2–4 nm). In addition, HMMINs also exhibited good magnetic properties, thermal stability, large adsorption capacity (37.64 ± 1.36 μmol/g), and fast kinetics (within 45 min) toward SMZ from pure water. Surface grafting of hydrophilic polymer brushes enhanced adsorption selectivity and kinetics rate. He et al. have produced magnetic organic-inorganic nanocomposites with ultrathin imprinted polymers through an *in situ* surface-initiated grafting technique from HNTs (He et al., 2016). This material possessed a fast dynamic property and an efficient imprinting effect owing to the thin surface-imprinted polymer layer.

Recently, polyhedral oligomeric silsesquioxanes (POSS) are ideal building blocks for constructing a variety of functional composites due to their nano-size and hybrid molecular frameworks; octavinyl POSS can be regarded as a promising imprinting platform due to their tailorability resulting from the residual vinyl groups (He et al., 2014). He et al. have proposed a nanomagnetic POSS-directing strategy to construct molecularly imprinted hybrid materials for enrofloxacin (ENR) determination in milk samples (He et al., 2014). The ENR imprinted material was facilely obtained through the copolymerization of active vinyl groups present on the surface of nanomagnetic POSS and functional monomer (methacrylic acid) binding with ENR. Moreover, a convenient method based on the ENR imprinted material combined with High-Performance Liquid Chromatography-UV detection was established for the simultaneous determination of three FQs from milk samples with average recoveries in the range of 75.6–108.9%. More importantly, the proposed imprinting technique on the surface of nanomagnetic POSS could generally be applicable to imprinting various organic molecules with bright prospects.

The application of certain special functional monomers in the synthesis of MMIPs has brought unprecedented stimuli-responsive MIPs. Xu et al. have successfully prepared new thermal-responsive MMIPs as a potential effective adsorbent for selectively removing SMZ existing in aquatic environments (Xu et al., 2012). Thermal-responsive MMIPs can be obtained by adopting γ-Fe_2_O_3_ nanoparticles as a magnetic substrate material, SMZ as a template, acrylamide as an assistant functional monomer, 2,2＇-azobisisobutyronitrile as an initiator, EGDMA as a crosslinking monomer, and N-isopropylacrylamide as a functional temperature-responsive monomer that made MMIPs swell and shrink in response to environmental temperatures. The obtained TMMIPs have good temperature response, selectivity, and reusability, providing the possibility for their application in antibiotics separation and controlled release. Huang et al. have obtained thermal-responsive MMIPs using N-isopropylacrylamide as a thermosensitive monomer. A reversible volume phase transition of temperature-responsive materials occurs when the temperature changes. This material showed good potential for future applications.

Furthermore, several emerging techniques (biosensor, chemiluminescence, etc.) based on MMIPs also offer new insights into antibiotic detection (Kim et al., 2010; Wang et al., 2014; Luo et al., 2015). A highly sensitive tetracycline (TC) sensor was developed based on a combination of MMIPs nanoparticles (Gao et al., 2019). To increase the sensitivity of the sensor, the MMIP was prepared by using Fe_3_O_4_ nanoparticles functionalized carboxyl group as support. The performance of the MMIP sensor based on SPR detection was investigated. When TC-captured MMIPs nanoparticles flowed over the surface of the SPR chip, the SPR signal significantly increased. In addition, one structurally high related analog (chlortetracycline) and two non-analog compounds (sulfadiazine and norfloxacin) were chosen as interference antibiotics to assess the ability of the SPR method. The results indicated that the MMIP sensor based on SPR detection can be employed to detect TC with high sensitivity and selectivity. The sensor is linearly in the 5.0–100 pg mL^−1^ TC concentration range with a low detection limit of 1.0 pg mL^−1^. The MMIPs nanoparticles served as both a probe for recognizing TC and a label to enhance the SPR signal.

Chemiluminescence is a highly sensitive analysis approach, with advantages of a simplified optical system, wide linear dynamic range, weak background light level, and consequently low detection limit (Mohammadi Toudeshki et al., 2018). Mohammadi Toudeshki et al. have reported a rapid and selective magnetic solid-phase microextraction method using MMIPs combined with chemiluminescence for the preconcentration and determination of furazolidone (Mohammadi Toudeshki et al., 2018). MMIPs were prepared using the SiO_2_-coated Fe_3_O_4_ nanoparticles as the magnetic supporter, furazolidone as the template, 4-vinylpyridine as the functional monomer, EGDMA as the crosslinker, and toluene as the porogen solvent. The synthesized MMIPs showed high sorption capacity (49.6 mg/g) with a detection limit of 0.027 μg/L.

Based on the outstanding ability of MMIPs to recognize antibiotics, this material can be used to separate other contaminants in different complex systems such as the human body, food, and aquatic environment. The wide range of samples and contaminants makes detection much more difficult, while MMIPs can stand out. MMIPs can effectively reduce matrix interference to realize the detection of low concentration pollutants. This material is a great boon to environmental pollution. In addition to realizing the importance of MMIPs in addressing the problem of contaminants, we should further think about the application possibilities of MMIPs in the future, for example, exploring whether MMIPs can be used as a platform for controlled drug release to deliver anticancer drugs; whether it is possible to identify multiple target analytes at the same time; whether more template molecules can be used in the preparation of MMIPs.

## Conclusion and Perspective

Antibiotic residues are a major obstacle to safeguarding human health and environmental safety. Establishing efficient and sensitive antibiotic detection methods is of great significance for the effective monitoring of antibiotic residues in food and environment samples. However, due to the complexity of samples and the variety of antibiotics, it is usually necessary to utilize adsorbents for effective sample pretreatment procedures before detection. MMIPs can be considered the perfect candidate for sample separation due to their excellent specific selectivity and magnetic sensitivity. This article discusses the application of MMIPs in the detection of antibiotics, including the techniques for synthesizing MMIPs and their innovative applied cases in antibiotics separation. Compared with traditional sample separation methods (solvent extraction, centrifugation, solid-phase extraction purification, etc.), MMIPs are employed for sample separation with high speed and low solvent consumption. In addition, MMIPs offer advantages of large specific surface area, outstanding selectivity, high adsorption capacity, and specific extraction performance for target compounds. The application of MMIPs has opened up a new window for analyzing multiple components in complex environmental samples.

## Glossary

MMIPs: magnetic molecularly imprinted polymers
MIT: molecular imprinting technology
MNPs: magnetic nanoparticles
PEG: polyethylene glycol
PVA: polyvinyl alcohol
PEI: polyethyleneimine
PVP: polyvinylpyrrolidone
PDA: polydopamine
PADs: polyamidoamine dendrimers
MIONPs: magnetic iron oxide nanoparticles
IONPs: iron oxide nanoparticles
MRI: magnetic resonance imaging
RGDS: fibronectin-derived arginine-glycine-aspartic acid-serine
Fe_3_O_4_@G_2_-PAD: second-generation PADs functionalized magnetic nanoparticles
MCPE: magnetic carbon paste electrode
O/W: oil-in-water
W/O: water-in-oil
MMA: methyl methacrylate
EGDMA: ethylene glycol dimethacrylate
AIBN: 2,2-azobis (2-methylpropionitrile)
CRP: controlled radical polymerization
ATRP: atom transfer radical polymerization
RAFT: reversible addition-fragmentation chain transfer
SMZ: sulfamethazine
EDA@Mag-CNTs-MIP: ethylenediamine-functionalized magnetic carbon nanotubes
FQs: fluoroquinolones
CNTs: carbon nanotubes
HNTs: halloysite nanotubes
HMMINs: hydrophilic magnetic surface molecularly imprinted core-shell nanorods
POSS: polyhedral oligomeric silsesquioxanes
ENR: enrofloxacin
TC: tetracycline
SPR: surface plasmon resonance
EM: erythromycin
CMS: carboxymethyl starch
MMT: montmorillonite.
